# Electronic Currents Induced by Optical Fields and Rotatory Power Density in Chiral Molecules

**DOI:** 10.3390/molecules26144195

**Published:** 2021-07-10

**Authors:** Francesco Ferdinando Summa, Guglielmo Monaco, Riccardo Zanasi, Stefano Pelloni, Paolo Lazzeretti

**Affiliations:** 1 Dipartimento di Chimica e Biologia “A. Zambelli”, Università Degli Studi di Salerno, via Giovanni Paolo II 132, 84084 Fisciano, Italy; fsumma@unisa.it (F.F.S.); gmonaco@unisa.it (G.M.); rzanasi@unisa.it (R.Z.); 2 Istituto d’Istruzione Superiore Francesco Selmi, via Leonardo da Vinci, 300, 41126 Modena, Italy; pelloni.stefano.69@gmail.com

**Keywords:** optical activity, rotatory power, electric dipole-magnetic dipole polarizability, spatial density functions of molecular response tensors, electronic current densities, translational invariance of computed properties, specific rotation

## Abstract

The electric dipole–magnetic dipole polarizability tensor $\kappa^{\prime }$, introduced to interpret the optical activity of chiral molecules, has been expressed in terms of a series of density functions $k_{\alpha \beta }^{\prime }$, which can be integrated all over the three-dimensional space to evaluate components $\kappa_{\alpha \beta }^{\prime }$ and trace $\kappa_{\alpha \alpha }^{\prime }$. A computational approach to $k_{\alpha \beta }^{\prime }$, based on frequency-dependent electronic current densities induced by monochromatic light shining on a probe molecule, has been developed. The dependence of $k_{\alpha \beta }^{\prime }$ on the origin of the coordinate system has been investigated in connection with the corresponding change of $\kappa_{\alpha \beta }^{\prime }$. It is shown that only the trace $k_{\alpha \alpha }^{\prime }$ of the density function defined via dynamic current density evaluated using the continuous translation of the origin of the coordinate system is invariant of the origin. Accordingly, this function is recommended as a tool that is quite useful for determining the molecular domains that determine optical activity to a major extent. A series of computations on the hydrogen peroxide molecule, for a number of different HO–OH dihedral angles, is shown to provide a pictorial documentation of the proposed method.

## 1. Preface

The sentence of Louis Pasteur, “L’univers est dissymétrique”, p. 4 of Ref. [1], is a milestone in the history of science. His concept of cosmic dissymmetry, ubiquitous at all levels of magnitude and characterizing every shape, for instance, “la forme général d’une hélice, d’une escalier tournant, d’une tétrahèdre irrégulaire, d’une main, d’une oeil...” [1], paved the way to modern quantum field theories and to the notion of Nature’s fundamental symmetries. Haldane argued that Pasteur’s prophetic conjecture provides an anticipation of the discovery of parity nonconserving forces [2].

Actually, the fundamental importance of the Pasteur conception, from both physical and philosophical points of view, relies on the fact that he “attributed the asymmetry to the universe, and not to a ‘vital force’ or some such agency acting in parts of it” [2].

The term “dissymmetry” was replaced by “chirality”, widely adopted nowadays and coined by William Thomson, Lord Kelvin, who, in his Baltimore Lectures [3], gave a strictly geometrical definition, “I call any geometrical figure, or group of points, chiral, and say that it has chirality if its image in a plane mirror, ideally realized, cannot be brought to coincide with itself”.

Notwithstanding, the idea of chirality acquired a much more general connotation and is commonly adopted within a wider acceptation. For instance, the notion of axial chirality has been adopted for systems not endowed with a stereogenic center, thus implying stereoisomerism that results from the nonplanar arrangement of substituent groups. In addition, concepts of various types of topological chirality have been advanced [4,5,6,7,8].

A particularly appealing topic is related to the rationalization of various mechanisms concurring to determine optical activity and to the search of powerful interpretative methods. Thus, the present chapter develops some novel ideas and, more specifically, the concept of density functions suitable for investigating the optical rotatory power of chiral molecules and related computational techniques. Pauling made two main contributions to the field of chiral species which have fundamental importance for living beings [9]. In 1936 he formulated a model for the structure of hemoglobin, in which atoms were arranged in chiral helical patterns [10]. Later on, he applied this idea to proteins in general, thus proposing that deoxyribonucleic acid (DNA) was a triple helix [11].

## 2. Introduction

If a beam of plane-polarized light is rotated upon passage through a medium, the substance that constitutes it is said to be optically active, meaning that it is endowed with optical rotatory power [12]. In the isotropic phase, the theory of optical activity attested by quantum mechanical methods of molecular physics [13,14,15] describes the phenomenology in terms of the mean electric dipole induced by the time-derivative of the magnetic field of the impinging light beam, and the mean magnetic dipole induced by the time-derivative of the associated electric field [16,17,18,19].

If the beam of light propagates in the *z* direction through a medium, anisotropic and symmetric about the *z* axis, as in a strong static electric field [20] or in a nematic liquid crystal in a field in the *k* direction, the optical activity of the oriented molecules is eminently anisotropic, determining different components along different directions. Moreover, as recognized early by Stephens, the neglect of electric–quadrupole terms can produce meaningless results [21].

In fact, within the electric–quadrupole approximation [22], the terms of the interaction Hamiltonian coupling magnetic dipole to the magnetic field—and electric quadrupole to the electric field gradient—have the same magnitude, as they result from the same order in the expansion of the vector potential. In particular, the expressions defining measurable properties are in general origin-invariant only when both terms are included [17,23,24].

Optical activity depends on the distribution of polarizable electronic domains within a chiral molecule. Thus, a helical arrangement of atoms, determining a particular type of polarizability, regulates the molecular response that leads to optical activity [18,25], which is caused by electronic currents flowing along helical paths [15,26,27].

Although this model may appear somewhat naive, or even factitious, it conveys the basic idea that epitomizes the type of electronic motion that determines optical rotatory power, that is, “the movement of electric charge along crooked pathways under the influence of light within the molecule” [26].

One may ask whether different regions of a chiral molecule are involved to the same extent in determining its optical activity, since effects that come into play in certain domains, for example, that containing a stereogenic center, could plausibly provide major contributions. It is intuitive that a reliable answer should be attempted in terms of some kind of density function, depending on a position coordinate, *r*, for any transparent frequency of the monochromatic radiation shining on the molecule. Such a density function would also enable the role of different substituents and chromophores to be investigated, alongside the enhancement or decrease of optical activity caused by various moieties via an auxochromic effect.

The overall features of a suitable “optical activity density function” are easily guessed a priori for isotropic media. As for the “nuclear magnetic shielding density” proposed by Jameson and Buckingham [28,29], it should be a second-rank tensor function of r, whose space integral provides the components of mixed electric dipole–magnetic dipole polarizability (MEMDP), introduced in Section 3 to rationalize its phenomenology [16,18,23].

In an ordered phase, for example, in a nematic liquid crystal, one should also take into account the contributions arising from mixed electric dipole electric quadrupole polarizability (MEDEQP) [17,23] and the corresponding property density.

At any rate, a basic requirement for reliable density functions, which may be visualized by plotting representations on a plane or via perspective three-dimensional maps, is that of origin independence [28,29]. The present study, focusing mainly on molecular response in isotropic media, aims to test practical definitions of MEMDP density, which can be easily implemented in computer packages currently available in order to assess to what extent they attain the demand of translational invariance.

The structure of this paper is as follows. The notation employed is outlined in Section 3. A definition of an MEMDP tensor based on two equivalent relationships that correspond to dipole length and dipole velocity gauges, and a series of MEMDP densities, are examined in Section 4, relying on different charge and current density functions, whose behaviours in a coordinate translation are discussed in Section 5. Methods employing the continuous translation of the origin of magnetically induced current density, taken into account in Section 6, are shown to fully satisfy the origin-independence prerequisite of MEMDP density needed to investigate optical activity in isotropic media. They also appear quite promising in view of further studies on the optical rotatory power of anisotropic samples. The implementation of theoretical methods is outlined in Section 7 and an application to hydrogen peroxide, chosen as a preliminary model system for studying axial chirality and demonstrating the practicality of the MEMDP density concept, is described in Section 8. Concluding remarks and outlook are reported in Section 9.

## 3. Outline of Notation and Theoretical Methods

Within the Born–Oppenheimer (BO) approximation [30], for a molecule with *n* electrons and *N* clamped nuclei, charge, mass, position, canonical and angular momentum of the *k*-th electron are indicated, in the configuration space, by −e, $m_{e}$, $r_{k}$, ${\hat{p}}_{k}=-i\hbar \nabla_{k}$, ${\hat{l}}_{k}=r_{k}\times {\hat{p}}_{k}$, k=1,2…n, using boldface letters for electronic vector operators. Analogous quantities for nucleus *I* are $Z_{I}e$, $M_{I}$, $R_{I}$, *etc.*, for I=1,2…N.

The imaginary unit is represented by a Roman i. Throughout this chapter, SI units are used and standard tensor formalism is employed, for example, the Einstein convention of implicit summation over two repeated Greek indices is in force. The third-rank Levi–Civita pseudotensor is indicated by $\epsilon_{\alpha \beta \gamma }$.

Capitals denote *n*-electron operators, for example, for position, canonical and angular momentum,

$$\hat{R}=\sum_{k=1}^{n}r_{k},\;\hat{P}=\sum_{k=1}^{n}{\hat{p}}_{k},\;\hat{L}=\sum_{k=1}^{n}{\hat{l}}_{k},$$

so the electric and magnetic dipole operators become

(1) $$\begin{array}{ccc} \hat{\mu } & = & -e\hat{R}, \end{array}$$

(2) $$\begin{array}{ccc} \hat{m} & = & -\frac{e}{2m_{e}}\hat{L}. \end{array}$$

Expressions for the polarization charge density and current density induced in the electrons of a molecule by optical fields are obtained by time-dependent quantum mechanical perturbation theory [31], assuming that the eigenvalue problem for the time-independent BO electronic Hamiltonian ${\hat{H}}^{\left(0\right)}\Psi_{j}^{\left(0\right)}=E_{j}^{\left(0\right)}\Psi_{j}^{\left(0\right)}$ has been solved, determining a set of eigenfunctions $\Psi_{j}^{\left(0\right)}$ and corresponding energy eigenvalues $E_{j}^{\left(0\right)}.$ The reference (ground) state is indicated by $\Psi_{a}^{\left(0\right)}$ and the natural transition frequencies are $\omega_{ja}=\left(E_{j}^{\left(0\right)}-E_{a}^{\left(0\right)}\right)/\hbar$.

The *n*-electron density matrix, expressed in the general form

(3) $$\gamma \left(x_{1};x_{1}^{\prime }\right)=n\int \Psi \left(x_{1},X_{1}\right)\Psi^{*}\left(x_{1}^{\prime },X_{1}\right)dX_{1},$$

within the McWeeny normalization [32], depends on electronic space–spin coordinates, $x_{k}=r_{k}⊗s_{k},\;k=1,2,\ldots ,n,$ where

(4) $$X_{1}\equiv \left\{x_{2},\ldots ,x_{n}\right\},\;X=\left\{x_{1},X_{1}\right\},\;dX_{1}\equiv \left\{dx_{2},\ldots ,dx_{n}\right\}.$$

Integrating over $ds_{1}$, one gets from Equation (3),

(5) $$\begin{array}{ccc} \gamma^{\left(0\right)}\left(r\right) & \equiv & \gamma^{\left(0\right)}\left(r;r\right)\\ & = & n\int \Psi_{a}^{\left(0\right)}\left(r,X_{1}\right)\Psi_{a}^{(0)*}\left(r,X_{1}\right)dX_{1} \end{array}$$

for the reference (ground) state $\Psi_{a}^{\left(0\right)}$ of the molecule, thus $\rho^{\left(0\right)}\left(r\right)=-e\gamma^{\left(0\right)}\left(r\right)$ is the electronic charge density in the absence of perturbation.

The probability current density [32] is obtained from Equations (3)–(5) for the density matrix,

(6) $$j\left(r\right)=\frac{1}{m_{e}}ℜ{\left[\hat{\pi }\gamma \left(r;r^{\prime }\right)\right]}_{r^{\prime }=r}\;.$$

In this equation one puts $r^{\prime }=r$ after operating with the electronic mechanical momentum,

(7) $$\hat{\pi }=\hat{p}+eA,$$

adopting the Bloch gauge [33] for the vector potential ***A***. The electron current density corresponding to (6) is obtained, multiplying by −e, that is, J=−ej. The interaction Hamiltonian considered in the present work does not contain terms depending on electron spin, therefore the probability current density (6) includes only orbital contributions.

To account for the magnetic response of a molecule to a time-dependent electromagnetic field, which, for the sake of simplicity, is represented by a monochromatic plane wave with frequency ω, the long-wavelength assumption [34,35] is relaxed, which amounts to postulating, within the next-higher electric quadrupole approximation [22], that the time-dependent electric field E(t) is not spatially uniform, whereas both the magnetic field B(t) and the electric field gradient ∇E(t) are homogeneous over the molecular dimensions.

If the intensity of B(t) is weak enough, first-order time-dependent perturbation theory [31] can be applied to describe the interacting system [36]. For instance, the total electronic charge density can be expressed as a truncated series,

(8) $$\rho \left(r\right)=\rho^{\left(0\right)}\left(r\right)+\rho^{\left(1\right)}\left(r\right)+\rho^{\left(2\right)}\left(r\right)+\cdots ,$$

introducing polarization densities of increasing order induced by the impinging wave. The magnetic field does not determine a first-order change in the diagonal terms of the density matrix (3), since the first-order perturbed electronic wavefunction is pure imaginary. On the other hand, the time derivative $\dot{B}\left(t\right)$ induces an oscillating polarization of the electronic distribution [22] so that, taking into account terms to first order in the magnetic field,

(9) $$\rho^{\left(1\right)}\left(r,\omega \right)\equiv \rho^{\dot{B}}\left(r,\omega \right)=\varrho^{{\dot{B}}_{\alpha }}\left(r,\omega \right){\dot{B}}_{\alpha }\left(t\right),$$

where

(10) $$\varrho^{{\dot{B}}_{\alpha }}\left(r,\omega \right)=\frac{\partial \rho^{\dot{B}}\left(r,\omega \right)}{\partial {\dot{B}}_{\alpha }}$$

is described by a vector function of position [37],

(11) $$\begin{array}{ccc} \varrho^{{\dot{B}}_{\alpha }}\left(r,\omega \right) & = & -\frac{2en}{\hbar }\sum_{j\neq a}{\left(\omega_{ja}^{2}-\omega^{2}\right)}^{-1}ℑ\left\{\langle a\left|{\hat{m}}_{\alpha }\right|j\rangle \right.\\ & & \left.\times \int \Psi_{j}^{(0)*}\left(r,X_{1}\right)\Psi_{a}^{\left(0\right)}\left(r,X_{1}\right)dX_{1}\right\}. \end{array}$$

Together with the polarization density defined by the scalar $\rho^{\dot{B}}\left(r,\omega \right)$, an electronic current density vector field $J^{B}\left(r,\omega \right)$ is induced by the oscillating optical field B(t) [36,37]. Its effects on molecular response are dealt with via practical computational procedures based on a dynamic, second-rank, current density tensor (CDT), obtained by differentiation [36,37],

(12) $$J_{\alpha }^{B_{\beta }}\left(r,\omega \right)=\frac{\partial J_{\alpha }^{B}\left(r,\omega \right)}{\partial B_{\beta }}.$$

It is expressed as a sum of paramagnetic and diamagnetic terms within the conventional common-origin (CO) assumption [38],

(13) $$\begin{array}{ccc} J_{\alpha }^{B_{\beta }}\left(r,\omega \right) & = & -\frac{ne}{m_{e}\hbar }\sum_{j\neq a}\frac{\omega_{ja}}{\omega_{ja}^{2}-\omega^{2}}\\ & & \times ℜ\left\{\langle a\left|{\hat{m}}_{\beta }\right|j\rangle \int \Psi_{j}^{(0)★}\left(r,X_{1}\right){\hat{p}}_{\alpha }\Psi_{a}^{\left(0\right)}\left(r,X_{1}\right)dX_{1}\right.\\ & & \left.+\int \Psi_{a}^{(0)★}\left(r,X_{1}\right){\hat{p}}_{\alpha }\Psi_{j}^{\left(0\right)}\left(r,X_{1}\right)dX_{1}\langle j\left|{\hat{m}}_{\beta }\right|a\rangle \right\}\\ & & -\frac{e^{2}}{2m_{e}}\epsilon_{\alpha \beta \gamma }r_{\gamma }\gamma^{\left(0\right)}\left(r\right). \end{array}$$

The time derivative of the electric field associated to the monochromatic light shining on the molecule induces an electronic current density [22] $J^{\dot{E}}\left(r,\omega \right)$. A corresponding CDT, to first order in the electric field, is obtained by differentiating,

(14) $$J_{\alpha }^{{\dot{E}}_{\beta }}\left(r,\omega \right)=\frac{\partial J_{\alpha }^{\dot{E}}\left(r,\omega \right)}{\partial {\dot{E}}_{\beta }}.$$

It is cast in the form [36]:

(15) $$\begin{array}{ccc} J_{\alpha }^{{\dot{E}}_{\beta }}\left(r,\omega \right) & = & -\frac{ne}{m_{e}\hbar }\sum_{j\neq a}{\left(\omega_{ja}^{2}-\omega^{2}\right)}^{-1}\\ & & \times ℑ\left\{\langle a\left|{\hat{\mu }}_{\beta }\right|j\rangle \int \Psi_{j}^{(0)★}\left(r,X_{1}\right){\hat{p}}_{\alpha }\Psi_{a}^{\left(0\right)}\left(r,X_{1}\right)dX_{1}\right.\\ & & \left.-\int \Psi_{a}^{(0)★}\left(r,X_{1}\right){\hat{p}}_{\alpha }\Psi_{j}^{\left(0\right)}\left(r,X_{1}\right)dX_{1}\langle j\left|{\hat{\mu }}_{\beta }\right|a\rangle \right\}. \end{array}$$

A useful connection between alternative approaches to the molecular response, allowing either for scalar polarization densities or vectorial current densities, is available within the framework of recent suggestions [36], introducing the interaction Lagrangian density and a perturbative expansion for the moments of the polarization charge density function ρ, in relation to corresponding moments of the current density *J*, via the general relationship [22,37]:

(16) $$\frac{d}{dt}\int r_{\alpha }\rho \;d^{3}r=\int J_{\alpha }d^{3}r.$$

In Section 4, we will make use of Equation (16), allowing for the definition of electronic charge density vector $\varrho^{{\dot{B}}_{\alpha }}\left(r,\omega \right)$, Equation (11), and current density tensor $J_{\alpha }^{B_{\beta }}\left(r,\omega \right)$, Equation (13).

## 4. Spatial Density of Electric Dipole-Magnetic Dipole Polarizability

To first order in $\dot{B}$, the electric dipole moment induced in the electron distribution is given by

(17) $$\Delta {\langle {\hat{\mu }}_{\alpha }\left(t\right)\rangle }^{\dot{B}}=\int r_{\alpha }\varrho^{{\dot{B}}_{\beta }}\left(r,\omega \right)d^{3}r\cdot {\dot{B}}_{\beta }\left(t\right)\equiv \kappa_{\alpha \beta }^{\prime }\left(\omega \right){\dot{B}}_{\beta }\left(t\right)\omega^{-1},$$

identifying the integral with the second-rank tensor,

(18) $$\begin{array}{ccc} \kappa_{\alpha \beta }^{\prime }\left(\omega \right) & = & -\frac{e^{2}}{2m_{e}\hbar }\sum_{j\neq a}\frac{2\omega }{\omega_{ja}^{2}-\omega^{2}}ℑ\left(\langle a\left|{\hat{R}}_{\alpha }\right|j\rangle \langle j\left|{\hat{L}}_{\beta }\right|a\rangle \right)\\ & = & -\frac{e^{2}}{2m_{e}^{2}\hbar }\sum_{j\neq a}\frac{2\omega }{\omega_{ja}\left(\omega_{ja}^{2}-\omega^{2}\right)}ℜ\left(\langle a\left|{\hat{P}}_{\alpha }\right|j\rangle \langle j\left|{\hat{L}}_{\beta }\right|a\rangle \right), \end{array}$$

that is, with the mixed electric dipole–magnetic dipole polarizability (MEMDP) in the dipole length-angular momentum (R,L) formalism, identical to that in the dipole velocity–angular momentum (P,L) formalism, if the off-diagonal hypervirial theorem [39,40],

(19) $$\langle a|\hat{R}|j\rangle =\frac{i}{m_{e}}\omega_{ja}^{-1}\langle a|\hat{P}|j\rangle ,$$

is satisfied [22,41]. Relationships (19) are obeyed by exact eigenfunctions to a model Hamiltonian and by optimal variational wavefunctions [39,40,42].

The trace of the MEMDP tensor (18) is related to the angle of natural optical rotation through the Rosenfeld equation [13,14]. In SI units,

(20) $$\phi =-\frac{1}{3}\omega \mu_{0}LN\kappa_{\alpha \alpha }^{\prime },$$

where $\mu_{0}$ is the permeability of free space, *L* is the path length, and N is the number density of optically active molecules [43].

The argument in the space integral (17) for the induced electric dipole moment can be used to define

(21) $$k_{\alpha \beta }^{\prime }\left(r,\omega \right)=\omega r_{\alpha }\varrho^{{\dot{B}}_{\beta }}\left(r,\omega \right),$$

that is, a second-rank tensor function of position *r*, nonsymmetric in the exchange α↔β of indices, referred to as the *electric dipole–magnetic dipole polarizability density* [37].

Using Equation (13), the off-diagonal hypervirial relationships (19), and the condition of completeness $\sum_{j\neq a}\left|j\rangle \langle j\right|=\hat{I}-\left|a\rangle \langle a\right|$, one finds [36,37]:

(22) $$\int J_{\alpha }^{B_{\beta }}\left(r,\omega \right)d^{3}r=-\omega \kappa_{\alpha \beta }^{\prime }\left(\omega \right).$$

This result, directly arrived at via definitions (12) and (13), and the r.h.s. of Equation (16), is fully consistent with the l.h.s. evaluated via (21), as $\ddot{B}=-\omega^{2}B$ for harmonic fields. Therefore, the function of position,

(23) $$k_{\alpha \beta }^{\prime }\left(r,\omega \right)=-\omega^{-1}J_{\alpha }^{B_{\beta }}\left(r,\omega \right),$$

defined via Equation (12), can also be interpreted as an electric dipole–magnetic dipole polarizability density function [37], alternative to (21).

A third expression for MEMDP density is arrived at from the contribution to the magnetic dipole moment induced in the electron cloud by the time derivative $\dot{E}$ of the electric field [37],

(24) $$\Delta {\langle {\hat{m}}_{\alpha }\left(t\right)\rangle }^{\dot{E}}=\frac{1}{2}\epsilon_{\alpha \beta \gamma }\int r_{\beta }J_{\gamma }^{{\dot{E}}_{\delta }}\;d^{3}r\cdot {\dot{E}}_{\delta }=-\kappa_{\delta \alpha }^{\prime }{\dot{E}}_{\delta }\;\omega^{-1},$$

and the CDT (14) and (15). Thus, according to Equation (24), another computational recipe for MEMDP density is given by

(25) $$k_{\delta \alpha }^{\prime }\left(r,\omega \right)=-\frac{1}{2}\omega \epsilon_{\alpha \beta \gamma }r_{\beta }J_{\gamma }^{{\dot{E}}_{\delta }}\left(r,\omega \right),$$

alternative to (21) and (23).

By identifying (23) with (25), one might inquire whether the relation

(26) $$J_{\delta }^{B_{\alpha }}\left(r,\omega \right)≊\frac{1}{2}\omega^{2}\epsilon_{\alpha \beta \gamma }r_{\beta }J_{\gamma }^{{\dot{E}}_{\delta }}\left(r,\omega \right)$$

is correct. In fact, using the off-diagonal hypervirial theorem (19), one finds

(27) $$\begin{array}{ccc} \frac{1}{2}\omega^{2}\epsilon_{\alpha \beta \gamma }r_{\beta }J_{\gamma }^{{\dot{E}}_{\delta }} & = & J_{d_{\delta }}^{B_{\alpha }}+\frac{ne^{2}}{2m_{e}^{2}\hbar }\sum_{j\neq a}\frac{\omega_{ja}}{\omega_{ja}^{2}-\omega^{2}}\\ & \times & ℜ\left\{\langle a\left|{\hat{P}}_{\delta }\right|j\rangle \int \Psi_{j}^{(0)*}{\hat{l}}_{\alpha }\Psi_{a}^{\left(0\right)}dX_{1}+\int \Psi_{a}^{(0)*}{\hat{l}}_{\alpha }\Psi_{j}^{\left(0\right)}dX_{1}\langle j\left|{\hat{P}}_{\delta }\right|a\rangle \right\}; \end{array}$$

thus, on comparing the r.h.s. of this equation with $J_{\delta }^{B_{\alpha }}\left(r,\omega \right)$, Equation (13), we can observe that only the diamagnetic contribution to the magnetic CDT is exactly recovered. The space integral of the second term on the r.h.s. of (27) is the same as that of the paramagnetic part $J_{p_{\delta }}^{B_{\alpha }}\left(r,\omega \right)$, defined via Equation (13). At any rate, the canonical and angular momentum operators are exchanged in (13) and (27), which implies that, in actual computations, employing finite basis sets, MEMDP densities (23) and (25) may be significantly different.

Further definitions of MEMDP density, which appear to be quite practical from the computational point of view, can directly be obtained from the second line of Equation (18), that is,

(28) $$\begin{array}{ccc} k_{\alpha \beta }^{\prime }\left(r,\omega \right) & = & -\frac{ne^{2}}{2m_{e}^{2}\hbar }\sum_{j\neq a}\frac{\omega }{\omega_{ja}\left(\omega_{ja}^{2}-\omega^{2}\right)}\\ & & \times ℜ\left\{\int \Psi_{a}^{(0)★}\left(r,X_{1}\right){\hat{p}}_{\alpha }\Psi_{j}^{\left(0\right)}\left(r,X_{1}\right)dX_{1}\langle j\left|{\hat{L}}_{\beta }\right|a\rangle \right. \end{array}$$

(29) $$\begin{array}{ccc} & & \left.+\langle a\left|{\hat{L}}_{\beta }\right|j\rangle \int \Psi_{j}^{(0)★}\left(r,X_{1}\right){\hat{p}}_{\alpha }\Psi_{a}^{\left(0\right)}\left(r,X_{1}\right)dX_{1}\right\},\\ k_{\alpha \beta }^{\prime }\left(r,\omega \right) & = & -\frac{ne^{2}}{2m_{e}^{2}\hbar }\sum_{j\neq a}\frac{\omega }{\omega_{ja}\left(\omega_{ja}^{2}-\omega^{2}\right)}\\ & & \times ℜ\left\{\langle a\left|{\hat{P}}_{\alpha }\right|j\rangle \int \Psi_{j}^{(0)★}\left(r,X_{1}\right){\hat{l}}_{\beta }\Psi_{a}^{\left(0\right)}\left(r,X_{1}\right)dX_{1}\right.\\ & & \left.+\int \Psi_{a}^{(0)★}\left(r,X_{1}\right){\hat{l}}_{\beta }\Psi_{j}^{\left(0\right)}\left(r,X_{1}\right)dX_{1}\langle j\left|{\hat{P}}_{\alpha }\right|a\rangle \right\}, \end{array}$$

and a symmetrized expression may be defined by one half of the sum of (28) and (29).

## 5. Origin Dependence of MEMDP and MEMDP Densities

It is easily verified that the integral of the polarization density vector (11), evaluated all over the molecular domain, vanishes due to the orthogonality of the eigenstates $\Psi_{a}^{\left(0\right)}$ and $\Psi_{j}^{\left(0\right)}$, thus fulfilling the constraint of charge conservation [37], that is,

(30) $$\int \rho^{\dot{B}}\left(r,\omega \right)d^{3}r=0;$$

however, the integral (17) for $\Delta {\langle {\hat{\mu }}_{\alpha }\left(t\right)\rangle }^{\dot{B}}$ is also a function of the *r* vector, whose components $r_{\alpha }$ depend on the origin $r^{\prime }$ chosen for the coordinate system, and change in a parallel translation represented by the arbitrary shift *d*,

(31) $$r^{\prime }\to r^{\prime \prime }=r^{\prime }+d.$$

An analogous statement is made for the integral (24), which defines the induced magnetic dipole moment $\Delta {\langle {\hat{m}}_{\alpha }\left(t\right)\rangle }^{\dot{E}}$. In fact, from Equation (15), one finds [37]

(32) $$\int J_{\alpha }^{{\dot{E}}_{\beta }}\left(r,\omega \right)d^{3}r=\alpha_{\beta \alpha }\left(\omega \right),$$

where $\alpha_{\beta \alpha }$ is the electric dipole polarizability in mixed dipole length–dipole velocity (R,P), identical to that in length (R,R) and velocity (P,P) pictures, according to corresponding definitions,

(33) $$\begin{array}{ccc} \alpha_{\alpha \beta }\left(\omega \right) & = & \frac{e^{2}}{m_{e}\hbar }\sum_{j\neq a}\frac{2}{\omega_{ja}^{2}-\omega^{2}}ℑ\left(\langle a\left|{\hat{R}}_{\alpha }\right|j\rangle \langle j\left|{\hat{P}}_{\beta }\right|a\rangle \right)\\ & = & \frac{e^{2}}{\hbar }\sum_{j\neq a}\frac{2\omega_{ja}}{\omega_{ja}^{2}-\omega^{2}}ℜ\left(\langle a\left|{\hat{R}}_{\alpha }\right|j\rangle \langle j\left|{\hat{R}}_{\beta }\right|a\rangle \right)\\ & = & \frac{e^{2}}{m_{e}^{2}\hbar }\sum_{j\neq a}\frac{2}{\omega_{ja}\left(\omega_{ja}^{2}-\omega^{2}\right)}ℜ\left(\langle a\left|{\hat{P}}_{\alpha }\right|j\rangle \langle j\left|{\hat{P}}_{\beta }\right|a\rangle \right), \end{array}$$

if the off-diagonal relations (19) are satisfied.

Therefore, the MEMDP densities (21) and (25) are expected to change in plots obtained using different coordinate systems. For this reason, visualizations of the tensor components of electric dipole–magnetic dipole polarizability density based on Equations (11), (17) and (21), or (24) and (25), as well as (28) and (29), are of doubtful physical meaning and are computationally impractical.

A more promising computational approach is possibly available via Equations (12) and (23). To test its main features, let us open a digression on the origin dependence of the MEMDP tensor (18). In the origin shift (31), the change of the angular momentum operator is given by

(34) $${\hat{L}}_{\beta }\left(r^{\prime \prime }\right)={\hat{L}}_{\beta }\left(r^{\prime }\right)-\epsilon_{\beta \gamma \delta }d_{\gamma }{\hat{P}}_{\delta },$$

thus the components defined by Equation (18) also change, while the trace remains the same,

(35) $$\kappa_{\alpha \beta }^{\prime }\left(r^{\prime \prime }\right)=\kappa_{\alpha \beta }^{\prime }\left(r^{\prime }\right)-\frac{\omega }{2}\epsilon_{\beta \gamma \delta }\alpha_{\alpha \gamma }d_{\delta },\;\mathrm{Tr}\left[\kappa^{\prime }\left(r^{\prime \prime }\right)\right]=\mathrm{Tr}\left[\kappa^{\prime }\left(r^{\prime }\right)\right].$$

According to Equation (35), the diagonal components of the MEMDP tensor are invariant of parallel translation if $\alpha_{\alpha \gamma }\left(\omega \right)$, symmetric under α↔γ, is referred to the principal axis system and accordingly represented by a diagonal matrix. In fact, the electric dipole polarizability frequently appears in diagonal form for symmetry reasons, for example, for molecules belonging to $D_{2}$, $D_{3}$, $D_{6}$, *T* and *O* point groups, in which off-diagonal components vanish. For molecules endowed with these symmetries, the principal axis system is easily guessed, for example, for $D_{2}$, by choosing $C_{2}^{\prime }\;\|\;x$ and $C_{2}^{\prime \prime }\;\|\;y$, for $D_{3}$ by choosing $C_{3}\;\|\;z$, $C_{2}\;\|\;x$, and so forth.

Within the algebraic approximation [44], if $\kappa_{\alpha \beta }^{\prime }$, Equation (18), is expressed either in (R,L) or in (P,L) pictures, thus $\alpha_{\alpha \gamma }$, Equation (33), is, respectively, expressed either in (R,P) or in (P,P) pictures in Equation (35). Accordingly, in actual computations, the trace of the MEMDP tensor calculated within the (P,L) picture via gaugeless basis sets is invariant in a translation of the coordinate system [22,45,46].

In any event, Equation (35) shows that the diagonal components of the MEMDP tensor (18), computed in (R,L) or in (P,L) pictures within the algebraic approximation [44], are invariant of the origin if, for a given value of the frequency ω, they are respectively referred to the coordinate system defined by the eigenvectors of the dynamic electric dipole polarizability (33) in (R,P) or (P,P) formalisms [41].

Now, although (R,R) and (P,P) electric polarizabilities are symmetric, that is, real Hermitian, and are always reducible to a principal axis system of orthogonal, that is, real unitary, eigenvectors, $\alpha_{\alpha \beta }$ computed within the (R,P) formalism may be slightly different from $\alpha_{\beta \alpha }$, unless the basis set is virtually complete, that is, big enough to guarantee that the hypervirial conditions (19) are satisfied to a good extent. In fact, on using small size basis sets, a polarizability tensor in mixed pictures, for example, length–velocity and velocity–acceleration [41], is, in the absence of molecular symmetry, represented by a nonsymmetric matrix which, in general, may have one real and two complex conjugate eigenvalues and nonorthogonal eigenvectors. This turns out to be a drawback which can be avoided by the use of extended basis sets.

Quite recently, newer methods, improving a procedure proposed for the (P,L) formalism [41] by applying a singular value decomposition of $\alpha_{\alpha \beta }$ in the (R,P) gauge, have been discussed to achieve origin invariant optical rotation in (R,L) formalism without London atomic orbitals [47,48,49]. Relations applicable to other gauges, including mixed dipole acceleration pictures reported elsewhere [41], could also be implemented allowing for singular value decomposition.

On the other hand, the magnetic CDT, Equation (12), changes according to

(36) $$J_{\alpha }^{B_{\beta }}\left(r,\omega \right)\to J_{\alpha }^{B_{\beta }}\left(r,\omega \right)+\frac{1}{2}\omega^{2}\epsilon_{\beta \gamma \delta }d_{\delta }J_{\alpha }^{{\dot{E}}_{\gamma }}\left(r,\omega \right),$$

in a translation of coordinate system (31). On account of Equation (32), Equation (36) is consistent with (35). It shows that the MEMDP density (23) depends on the origin. However, for computational purposes, the trace,

(37) $$k_{\alpha \alpha }^{\prime }=-\omega^{-1}J_{\alpha }^{B_{\alpha }}\left(r,\omega \right)$$

would seem preferable to those provided by (21) or (25) in that, according to Equation (35),

$$\kappa_{\alpha \alpha }^{\prime }=-\omega^{-1}\int J_{\alpha }^{B_{\alpha }}d^{3}r$$

would remain invariant in the translation (31).

An estimate of origin independence for $k_{\alpha \alpha }^{\prime }$, Equation (37), and $\kappa_{\alpha \alpha }^{\prime }$, is given for any direction δ by the modulus of vector

$$\epsilon_{\alpha \gamma \delta }\int J_{\alpha }^{{\dot{E}}_{\gamma }}\left(r,\omega \right)d^{3}r\approx 0,$$

allowing for Equations (35) and (36). It is expected to vanish identically in the ideal case of computations using complete basis sets [44], which would fulfill the hypervirial relationship (19). For truncated basis sets, the degree to which this integral approaches zero yields a measure of translational invariance for $k_{\alpha \alpha }^{\prime }$ densities estimated by Equation (23) and basis set quality. A pointwise test of symmetry $\left|J_{\alpha }^{{\dot{E}}_{\gamma }}\left(r,\omega \right)-J_{\gamma }^{{\dot{E}}_{\alpha }}\left(r,\omega \right)\right|<\epsilon$ of the integrand function, for a given positive threshold ϵ, would provide exhaustive indications.

Eventually, let us see if definitions (28) and (29) of MEMDP densities may yield recommendable computational recipes as regards the requisite of origin independence.

In fact, for any gaugeless basis set, the trace of MEMDP within the (P,L) formalism is origin independent according to (34) and (35). In the parallel translation (31), the change of the MEMDP densities (28) and (29) in the (P,L) picture is obtained respectively from

(38) $$\begin{array}{ccc} k_{\alpha \beta }^{\prime }\left(r-r^{\prime \prime },\omega \right) & = & k_{\alpha \beta }^{\prime }\left(r-r^{\prime },\omega \right)+\frac{\omega }{2}\epsilon_{\beta \gamma \delta }d_{\gamma }J_{\alpha }^{{\dot{E}}_{\delta }}\left(r,\omega \right), \end{array}$$

(39) $$\begin{array}{ccc} k_{\alpha \beta }^{\prime }\left(r-r^{\prime \prime },\omega \right) & = & k_{\alpha \beta }^{\prime }\left(r-r^{\prime },\omega \right)+\frac{\omega }{2}\epsilon_{\beta \gamma \delta }d_{\gamma }J_{\delta }^{{\dot{E}}_{\alpha }}\left(r,\omega \right), \end{array}$$

where the CDT (15) is re-expressed within the dipole velocity formalism via the hypervirial relation (19), that is, via the function:

(40) $$\begin{array}{ccc} J_{\alpha }^{{\dot{E}}_{\beta }}\left(r,\omega \right) & = & \frac{ne^{2}}{m_{e}^{2}\hbar }\sum_{j\neq a}{\left[\omega_{ja}\left(\omega_{ja}^{2}-\omega^{2}\right)\right]}^{-1}\\ & & \times ℜ\left\{\langle a\left|{\hat{P}}_{\beta }\right|j\rangle \int \Psi_{j}^{(0)★}\left(r,X_{1}\right){\hat{p}}_{\alpha }\Psi_{a}^{\left(0\right)}\left(r,X_{1}\right)dX_{1}\right.\\ & & \left.+\int \Psi_{a}^{(0)★}\left(r,X_{1}\right){\hat{p}}_{\alpha }\Psi_{j}^{\left(0\right)}\left(r,X_{1}\right)dX_{1}\langle j\left|{\hat{P}}_{\beta }\right|a\rangle \right\}. \end{array}$$

Therefore, within the (P,L) formalism, one finds from Equation (39),

(41) $$\kappa_{\alpha \beta }^{\prime }\left(r^{\prime \prime }\right)=\kappa_{\alpha \beta }^{\prime }\left(r^{\prime }\right)+\frac{\omega }{2}\epsilon_{\beta \gamma \delta }d_{\gamma }\int J_{\alpha }^{{\dot{E}}_{\delta }}\left(r,\omega \right)d^{3}r,$$

on account of (32) and (40). Thus the trace $\kappa_{\alpha \alpha }^{\prime }$ is identical to the second line of (18) and invariant of the origin for any gaugeless basis set if the MEMDP density (28) is used. However, even if the second term on the r.h.s. of Equation (38) vanishes on integrating over the whole space, thus obtaining the second term on the r.h.s. of (41), the density $k_{\alpha \alpha }^{\prime }$ defined by (28) is not expected to be invariant of the origin. The same result is arrived at via Equation (39).

## 6. Methods Employing Continuous Translation of the Origin of Magnetically Induced Current Density

A different expression for MEMDP density is arrived at within the approach referred to as CTOCD–DZ, whereby the CO diamagnetic term of Equation (13) is formally set to zero via a continuous translation of the origin [38]. Analogous results have been obtained via approaches using propagator methods and current density functional theory by Raimbault and coworkers [50,51].

The relationship defining the total CTOCD–DZ current density contains two non-Larmor terms, both referred to the same coordinate system, whose origin is not specified,

(42) $$J^{\mathrm{DZ}}\left(r,\omega \right)=J_{p}^{B}\left(r,\omega \right)+J_{p}^{r\times B}\left(r,\omega \right),$$

since their sum is invariant in a translation of the coordinate system [36]. In fact, the CO diamagnetic part of Equation (13) is replaced by the formally paramagnetic term,

(43) $$\begin{array}{ccc} J_{p_{\alpha }}^{r\times B}\left(r,\omega \right) & = & -\frac{ne^{2}}{2m_{e}^{2}\hbar }\epsilon_{\beta \gamma \delta }r_{\gamma }B_{\beta }\left(t\right)\sum_{j\neq a}\frac{\omega_{ja}}{\omega_{ja}^{2}-\omega^{2}}\\ & & \times ℜ\left\{\langle a\left|{\hat{P}}_{\delta }\right|j\rangle \int \Psi_{j}^{(0)*}\left(r,X_{1}\right){\hat{p}}_{\alpha }\right.\Psi_{a}^{\left(0\right)}\left(r,X_{1}\right)dX_{1}\\ & & +\int \Psi_{a}^{(0)*}\left(r,X_{1}\right){\hat{p}}_{\alpha }\left.\Psi_{j}^{\left(0\right)}\left(r,X_{1}\right)dX_{1}\langle j\left|{\hat{P}}_{\delta }\right|a\rangle \right\}, \end{array}$$

such that

(44) $$\begin{array}{c} J_{p_{\alpha }}^{r\times B}\left(r,\omega \right)=J_{d_{\alpha }}^{B}\left(r,\omega \right)+\Delta_{\alpha }\left(r,\omega \right), \end{array}$$

where

(45) $$\begin{array}{ccc} \Delta_{\alpha }\left(r,\omega \right) & = & \frac{ine^{2}}{2m_{e}\hbar }\epsilon_{\beta \gamma \delta }r_{\gamma }B_{\beta }\left(t\right)\sum_{j\neq a}\frac{\omega^{2}}{\omega_{ja}^{2}-\omega^{2}}\\ & & \times ℜ\left\{\langle a|{\hat{R}}_{\delta }\lfloor j\rangle \int \Psi_{j}^{(0)*}\left(r,X_{1}\right){\hat{p}}_{\alpha }\right.\Psi_{a}^{\left(0\right)}\left(r,X_{1}\right)dX_{1}\\ & & -\int \Psi_{a}^{(0)*}\left(r,X_{1}\right){\hat{p}}_{\alpha }\left.\Psi_{j}^{\left(0\right)}\left(r,X_{1}\right)dX_{1}\langle j\left|{\hat{r}}_{\delta }\right|a\rangle \right\}, \end{array}$$

if the off-diagonal theorem (19) is satisfied [36]. It is observed that the ω-dependent term (43) is obtained from the contribution to the static CTOCD–DZ current density [38] via the simple replacement,

$$\frac{1}{\omega_{ja}}\to \frac{\omega_{ja}}{\omega_{ja}^{2}-\omega^{2}}.$$

The CDT corresponding to the CTOCD–DZ current density (42), obtained by differentiating the current density (42),

(46) $$I_{\alpha }^{B_{\beta }}\left(r,\omega \right)=\frac{\partial J_{\alpha }^{\mathrm{DZ}}\left(r,\omega \right)}{\partial B_{\beta }},$$

defines a density function independent of the origin of the coordinate system. The corresponding space integral,

(47) $$\int I_{\alpha }^{B_{\beta }}\left(r,\omega \right)d^{3}r=-\frac{1}{2}\omega \left[\kappa_{\gamma \gamma }^{\prime }\delta_{\alpha \beta }+\kappa_{\alpha \beta }^{\prime }+\omega \epsilon_{\beta \gamma \delta }\alpha_{\delta ,\gamma \alpha }\right],$$

yields an origin-independent sum of terms [36] including a third-rank tensor, which represents the mixed electric dipole–electric quadrupole polarizability,

(48) $$\alpha_{\alpha ,\beta \gamma }=\frac{1}{\hbar }\sum_{j\neq a}\frac{2\omega_{ja}}{\omega_{ja}^{2}-\omega^{2}}ℜ\left(\langle a\left|{\hat{\mu }}_{\alpha }\right|j\rangle \langle j\left|{\hat{\mu }}_{\beta \gamma }\right|a\rangle \right),$$

where the electronic operator for the electric quadrupole in the Bloch gauge [33] is

(49) $${\hat{\mu }}_{\alpha \beta }=-\frac{e}{2}\sum_{k=1}^{n}{\left(r_{\alpha }r_{\beta }\right)}_{k}.$$

Owing to translational invariance of the CTOCD–DZ current density (42), and corresponding CDT (46), the trace of CTOCD–DZ MEMDP density,

(50) $$k_{\alpha \alpha }^{\prime }\left(r,\omega \right)=-\frac{1}{2\omega }I_{\alpha }^{B_{\alpha }}\left(r,\omega \right),$$

is also invariant for any *r* all over the molecular domain. Therefore, it provides the origin-independent MEMDP density required for computational purposes, exactly satisfying Equation (35), that is, $\kappa_{\alpha \alpha }^{\prime }\left(r^{\prime \prime }\right)=\kappa_{\alpha \alpha }^{\prime }\left(r^{\prime }\right)$.

The origin independence of the integral (47) is easily proven via Equation (35) and the corresponding change of MEDEQP, Equation (48),

(51) $$\alpha_{\delta ,\gamma \alpha }\left(r^{\prime \prime }\right)=\alpha_{\delta ,\gamma \alpha }\left(r^{\prime }\right)-\frac{1}{2}\alpha_{\delta \gamma }d_{\alpha }-\frac{1}{2}\alpha_{\delta \alpha }d_{\gamma }.$$

There is an interesting connection between the integral (47) and an invariant of the origin, which can be expressed in the form [24],

(52) $$I_{xyz}=\omega \left(\alpha_{x,yz}-\alpha_{y,xz}\right)-\kappa_{zz}^{\prime },$$

proposed by Buckingham and Dunn in their investigation on the optical activity of an anisotropic sample of oriented molecules [17,23]. For the optical rotation per unit path length, that is, for L=1 m of plane polarized light propagating in the *z* direction, these authors obtained a generalization of the Rosenfeld Equation (20) [13,14],

(53) $$\phi =-\frac{1}{2}\omega \mu_{0}N\zeta_{xyz}^{\prime },$$

where

(54) $$\zeta_{xyz}^{\prime }=\kappa_{\alpha \alpha }^{\prime }-\kappa_{zz}^{\prime }+\omega \left(\alpha_{x,yz}-\alpha_{y,xz}\right)=\kappa_{\alpha \alpha }^{\prime }+I_{xyz}.$$

For a monochromatic wave propagating in the z direction, the integral (47) becomes

(55) $$\int I_{z}^{B_{z}}\left(r,\omega \right)d^{3}r=-\frac{1}{2\omega }\left(\kappa_{\alpha \alpha }^{\prime }-I_{xyz}\right).$$

This expression is independent of the choice of origin. Thus, the theory developed in Ref. [18] is applicable to investigating optical activity in oriented molecules.

## 7. Implementation

The theoretical formulation of the MEMDP density functions described in the previous section can be implemented within the random phase approximation (RPA) formulation of the TD–HF [52,53,54] and TD–DFT [55,56,57,58] frameworks. For this purpose, we substitute (11) in (21) and use (2) to obtain the MEMDP density function in the (r,L) formalism,

(56) $$\begin{array}{ccc} k_{\alpha \beta }^{\prime (r,L)}\left(r,\omega \right) & = & \frac{ne^{2}}{2m_{e}\hbar }\sum_{j\neq a}\frac{\omega }{\omega_{ja}^{2}-\omega^{2}}ℑ\left\{\langle a\left|{\hat{L}}_{\beta }\right|j\rangle \right.\\ & & \times \int \Psi_{j}^{(0)*}\left(r,X_{1}\right)r_{\alpha }\Psi_{a}^{\left(0\right)}\left(r,X_{1}\right)dX_{1}\\ & & \left.-\int \Psi_{a}^{(0)*}\left(r,X_{1}\right)r_{\alpha }\Psi_{j}^{\left(0\right)}\left(r,X_{1}\right)dX_{1}\langle j\left|{\hat{L}}_{\beta }\right|a\rangle \right\}, \end{array}$$

which has to be distinguished from the MEMDP density function in the (l,R) formalism,

(57) $$\begin{array}{cc} k_{\alpha \beta }^{\prime (l,R)}\left(r,\omega \right)= & \frac{ne^{2}}{2m_{e}\hbar }\sum_{j\neq a}\frac{\omega }{\omega_{ja}^{2}-\omega^{2}}ℑ\{\langle a\left|{\hat{R}}_{\beta }\right|j\rangle \\ \; & \times \int \Psi_{j}^{(0)*}\left(r,X_{1}\right){\hat{l}}_{\alpha }\Psi_{a}^{\left(0\right)}\left(r,X_{1}\right)dX_{1}\\ \; & -\int \Psi_{a}^{(0)*}\left(r,X_{1}\right){\hat{l}}_{\alpha }\Psi_{j}^{\left(0\right)}\left(r,X_{1}\right)dX_{1}\langle j\left|{\hat{R}}_{\beta }\right|a\rangle \}. \end{array}$$

As can be easily shown, allowing for the hypervirial relationship (19), Equation (58) transforms into the MEMDP density in the (l,P) formalism given in Equation (29), as well as the MEMDP density in the (p,L) formalism given in Equaiton (28). As we will show shortly, densities (28), (29), (56), and (57) are all different from one another, even if, upon integration, they yield the same MEMDP tensors, that is, the integration of (56) and (57) gives two transposed tensors; the same occurs integrating (28) and (29). The two pairs of transposed tensors became equal in the complete basis set limit.

For the sake of implementation, the previous densities are rewritten in the form:

(58) $$\begin{array}{cc} k_{\alpha \beta }^{\prime (r,L)}\left(r,\omega \right)= & -\frac{ne^{2}}{2m_{e}\hbar }\omega \;\;ℑ\{\int \Psi_{a}^{{\left(L_{\beta }\right)}_{0}*}\left(r,X_{1},\omega \right)r_{\alpha }\Psi_{a}^{\left(0\right)}\left(r,X_{1}\right)dX_{1}\\ \; & +\int \Psi_{a}^{(0)*}\left(r,X_{1}\right)r_{\alpha }\Psi_{a}^{{\left(L_{\beta }\right)}_{0}}\left(r,X_{1},\omega \right)dX_{1}\}, \end{array}$$

(59) $$\begin{array}{cc} k_{\alpha \beta }^{\prime (l,R)}\left(r,\omega \right)= & -\frac{ne^{2}}{2m_{e}\hbar }\omega \;\;ℑ\{\int \Psi_{a}^{{\left(R_{\beta }\right)}_{0}*}\left(r,X_{1},\omega \right){\hat{l}}_{\alpha }\Psi_{a}^{\left(0\right)}\left(r,X_{1}\right)dX_{1}\\ \; & -\int \Psi_{a}^{(0)*}\left(r,X_{1}\right){\hat{l}}_{\alpha }\Psi_{a}^{{\left(R_{\beta }\right)}_{0}}\left(r,X_{1},\omega \right)dX_{1}\}, \end{array}$$

(60) $$\begin{array}{cc} k_{\alpha \beta }^{\prime (p,L)}\left(r,\omega \right)= & -\frac{ne^{2}}{2m_{e}^{2}\hbar }\omega \;\;ℜ\{-\int \Psi_{a}^{{\left(L_{\beta }\right)}_{-1}*}\left(r,X_{1},\omega \right){\hat{p}}_{\alpha }\Psi_{a}^{\left(0\right)}\left(r,X_{1}\right)dX_{1}\\ \; & +\int \Psi_{a}^{(0)*}\left(r,X_{1}\right){\hat{p}}_{\alpha }\Psi_{a}^{{\left(L_{\beta }\right)}_{-1}}\left(r,X_{1},\omega \right)dX_{1}\}, \end{array}$$

(61) $$\begin{array}{cc} k_{\alpha \beta }^{\prime (l,P)}\left(r,\omega \right)= & -\frac{ne^{2}}{2m_{e}^{2}\hbar }\omega \;\;ℜ\{-\int \Psi_{a}^{{\left(P_{\beta }\right)}_{-1}*}\left(r,X_{1},\omega \right){\hat{l}}_{\alpha }\Psi_{a}^{\left(0\right)}\left(r,X_{1}\right)dX_{1}\\ \; & +\int \Psi_{a}^{(0)*}\left(r,X_{1}\right){\hat{l}}_{\alpha }\Psi_{a}^{{\left(P_{\beta }\right)}_{-1}}\left(r,X_{1},\omega \right)dX_{1}\}, \end{array}$$

where

$$\begin{array}{c} \Psi_{a}^{{\left(L_{\beta }\right)}_{0}}\left(r,X_{1},\omega \right)=\sum_{j\neq a}\frac{\langle j|{\hat{L}}_{\beta }|a\rangle }{\omega_{ja}^{2}-\omega^{2}}\Psi_{j}^{\left(0\right)}\left(r,X_{1}\right) \end{array}$$

$$\begin{array}{c} \Psi_{a}^{{\left(r_{\beta }\right)}_{0}}\left(r,X_{1},\omega \right)=\sum_{j\neq a}\frac{\langle j|{\hat{r}}_{\beta }|a\rangle }{\omega_{ja}^{2}-\omega^{2}}\Psi_{j}^{\left(0\right)}\left(r,X_{1}\right), \end{array}$$

$$\begin{array}{c} \Psi_{a}^{{\left(L_{\beta }\right)}_{-1}}\left(r,X_{1},\omega \right)=\sum_{j\neq a}\frac{\langle j|{\hat{L}}_{\beta }|a\rangle }{\omega_{ja}\left(\omega_{ja}^{2}-\omega^{2}\right)}\Psi_{j}^{\left(0\right)}\left(r,X_{1}\right), \end{array}$$

$$\begin{array}{c} \Psi_{a}^{{\left(P_{\beta }\right)}_{-1}}\left(r,X_{1},\omega \right)=\sum_{j\neq a}\frac{\langle j|{\hat{P}}_{\beta }|a\rangle }{\omega_{ja}\left(\omega_{ja}^{2}-\omega^{2}\right)}\Psi_{j}^{\left(0\right)}\left(r,X_{1}\right), \end{array}$$

can now be regarded as perturbed wavefunctions explicitly depending on the radiation frequency. In the same way, the current density tensor (13) can be conveniently rewritten as,

(62) $$\begin{array}{cc} J_{\alpha }^{B_{\beta }}\left(r,\omega \right)= & \frac{ne^{2}}{2m_{e}^{2}\hbar }ℜ\{\int -\Psi_{a}^{{\left(L_{\beta }\right)}_{+1}*}\left(r,X_{1},\omega \right){\hat{p}}_{\alpha }\Psi_{a}^{\left(0\right)}\left(r,X_{1}\right)dX_{1}\\ \; & +\int \Psi_{a}^{(0)*}\left(r,X_{1}\right){\hat{p}}_{\alpha }\Psi_{a}^{{\left(L_{\beta }\right)}_{+1}}\left(r,X_{1},\omega \right)dX_{1}\}\\ \; & -\frac{e^{2}}{2m_{e}}\epsilon_{\alpha \beta \gamma }r_{\gamma }\gamma^{\left(0\right)}\left(r\right), \end{array}$$

where

$$\begin{array}{c} \Psi_{a}^{{\left(L_{\beta }\right)}_{+1}}\left(r,X_{1},\omega \right)=\sum_{j\neq a}\frac{\omega_{ja}\langle j|{\hat{L}}_{\beta }|a\rangle }{\omega_{ja}^{2}-\omega^{2}}\Psi_{j}^{\left(0\right)}\left(r,X_{1}\right). \end{array}$$

Within the CTOCD–DZ approach, the diamagnetic contribution is replaced by the formally paramagnetic term,

(63) $$\begin{array}{cc} I_{p\alpha }^{{\left(d\times B\right)}_{\beta }}\left(r,\omega \right)= & \frac{ne^{2}}{2m_{e}^{2}\hbar }\epsilon_{\beta \gamma \delta }r_{\gamma }ℜ\{\int \Psi_{a}^{{\left(P_{\delta }\right)}_{+1}*}\left(r,X_{1},\omega \right){\hat{p}}_{\alpha }\Psi_{a}^{\left(0\right)}\left(r,X_{1}\right)dX_{1}\\ \; & +\int \Psi_{a}^{(0)*}\left(r,X_{1}\right){\hat{p}}_{\alpha }\Psi_{a}^{{\left(P_{\delta }\right)}_{+1}}\left(r,X_{1},\omega \right)dX_{1},\} \end{array}$$

where

$$\begin{array}{c} \Psi_{a}^{{\left(P_{\delta }\right)}_{+1}}\left(r,X_{1},\omega \right)=\sum_{j\neq a}\frac{\omega_{ja}\langle j|{\hat{P}}_{\delta }|a\rangle }{\omega_{ja}^{2}-\omega^{2}}\Psi_{j}^{\left(0\right)}\left(r,X_{1}\right). \end{array}$$

For a closed-shell system, in the one-determinant approximation, assuming real molecular orbitals, densities (58)–(63) take the form that has been coded in atomic units:

(64) $$k_{\alpha \beta }^{\prime (r,L)}\left(r,\omega \right)=\omega \sum_{i}^{\mathrm{occ}}\left[\psi_{i}^{{\left({\left[r\times \nabla \right]}_{\beta }\right)}_{0}}\left(r,\omega \right)r_{\alpha }\psi_{i}^{\left(0\right)}\left(r\right)+\psi_{i}^{\left(0\right)}\left(r\right)r_{\alpha }\psi_{i}^{{\left({\left[r\times \nabla \right]}_{\beta }\right)}_{0}}\left(r,\omega \right)\right]$$

(65) $$k_{\alpha \beta }^{\prime (l,R)}\left(r,\omega \right)=\omega \sum_{i}^{\mathrm{occ}}\left[\psi_{i}^{{\left(r_{\beta }\right)}_{0}}\left(r,\omega \right)\epsilon_{\alpha \gamma \delta }r_{\gamma }\nabla_{\delta }\psi_{i}^{\left(0\right)}\left(r\right)-\psi_{i}^{\left(0\right)}\left(r\right)\epsilon_{\alpha \gamma \delta }r_{\gamma }\nabla_{\delta }\psi_{i}^{{\left(r_{\beta }\right)}_{0}}\left(r,\omega \right)\right]$$

(66) $$k_{\alpha \beta }^{\prime (p,L)}\left(r,\omega \right)=\omega \sum_{i}^{\mathrm{occ}}\left[-\psi_{i}^{{\left({\left[r\times \nabla \right]}_{\beta }\right)}_{-1}}\left(r,\omega \right)\nabla_{\alpha }\psi_{i}^{\left(0\right)}\left(r\right)+\psi_{i}^{\left(0\right)}\left(r\right)\nabla_{\alpha }\psi_{i}^{{\left({\left[r\times \nabla \right]}_{\beta }\right)}_{-1}}\left(r,\omega \right)\right]$$

(67) $$k_{\alpha \beta }^{\prime (l,P)}\left(r,\omega \right)=\omega \sum_{i}^{\mathrm{occ}}\left[\psi_{i}^{{\left(\nabla_{\beta }\right)}_{-1}}\left(r,\omega \right)\epsilon_{\alpha \gamma \delta }r_{\gamma }\nabla_{\delta }\psi_{i}^{\left(0\right)}\left(r\right)-\psi_{i}^{\left(0\right)}\left(r\right)\epsilon_{\alpha \gamma \delta }r_{\gamma }\nabla_{\delta }\psi_{i}^{{\left(\nabla_{\beta }\right)}_{-1}}\left(r,\omega \right)\right]$$

(68) $$\begin{array}{cc} J_{\alpha }^{B_{\beta }}\left(r,\omega \right)\; & =\sum_{i}^{\mathrm{occ}}\left[\psi_{i}^{{\left({\left[r\times \nabla \right]}_{\beta }\right)}_{+1}}\left(r,\omega \right)\nabla_{\alpha }\psi_{i}^{\left(0\right)}\left(r\right)-\psi_{i}^{\left(0\right)}\left(r\right)\nabla_{\alpha }\psi_{i}^{{\left({\left[r\times \nabla \right]}_{\beta }\right)}_{+1}}\left(r,\omega \right)\right]\\ \; & -\epsilon_{\alpha \beta \gamma }r_{\gamma }\sum_{i}^{\mathrm{occ}}\psi_{i}^{\left(0\right)}\psi_{i}^{\left(0\right)} \end{array}$$

(69) $$\begin{array}{cc} I_{\alpha }^{B_{\beta }}\left(r,\omega \right) & =\sum_{i}^{\mathrm{occ}}\left[\psi_{i}^{{\left({\left[r\times \nabla \right]}_{\beta }\right)}_{+1}}\left(r,\omega \right)\nabla_{\alpha }\psi_{i}^{\left(0\right)}\left(r\right)-\psi_{i}^{\left(0\right)}\left(r\right)\nabla_{\alpha }\psi_{i}^{{\left({\left[r\times \nabla \right]}_{\beta }\right)}_{+1}}\left(r,\omega \right)\right]\\ \; & -\epsilon_{\alpha \beta \gamma }r_{\gamma }\sum_{i}^{\mathrm{occ}}\left[\psi_{i}^{(\nabla_{\delta })+1}\left(r,\omega \right)\nabla_{\alpha }\psi_{i}^{\left(0\right)}\left(r\right)-\psi_{i}^{\left(0\right)}\left(r\right)\nabla_{\alpha }\psi_{i}^{(\nabla_{\delta })+1}\left(r,\omega \right)\right] \end{array}$$

In the above equations and in the following, i,m indices denote occupied and virtual orbitals, respectively, and *q* is used for basis set functions. Molecular orbitals $\psi_{i}$ are expanded as linear combinations of basis set functions $\chi_{q}$,

(70) $$\begin{array}{cc} \psi_{i}^{\left(0\right)}\left(r\right)= & \sum_{q}C_{qi}^{\left(0\right)}\chi_{q}\left(r\right), \end{array}$$

(71) $$\begin{array}{cc} \psi_{i}^{{\left({\left[r\times \nabla \right]}_{\beta }\right)}_{0}}\left(r,\omega \right)= & \sum_{q}C_{qi}^{{\left({\left[r\times \nabla \right]}_{\beta }\right)}_{0}}\left(\omega \right)\chi_{q}\left(r\right) \end{array}$$

(72) $$\begin{array}{cc} \psi_{i}^{{\left(r_{\beta }\right)}_{0}}\left(r,\omega \right)= & \sum_{q}C_{qi}^{{\left(r_{\beta }\right)}_{0}}\left(\omega \right)\chi_{q}\left(r\right) \end{array}$$

(73) $$\begin{array}{cc} \psi_{i}^{{\left({\left[r\times \nabla \right]}_{\beta }\right)}_{-1}}\left(r,\omega \right)= & \sum_{q}C_{qi}^{{\left({\left[r\times \nabla \right]}_{\beta }\right)}_{-1}}\left(\omega \right)\chi_{q}\left(r\right) \end{array}$$

(74) $$\begin{array}{cc} \psi_{i}^{{\left(\nabla_{\beta }\right)}_{-1}}\left(r,\omega \right)= & \sum_{q}C_{qi}^{{\left(\nabla_{\beta }\right)}_{-1}}\left(\omega \right)\chi_{q}\left(r\right) \end{array}$$

(75) $$\begin{array}{cc} \psi_{i}^{{\left({\left[r\times \nabla \right]}_{\beta }\right)}_{+1}}\left(r,\omega \right)= & \sum_{q}C_{qi}^{{\left({\left[r\times \nabla \right]}_{\beta }\right)}_{+1}}\left(\omega \right)\chi_{q}\left(r\right) \end{array}$$

(76) $$\begin{array}{cc} \psi_{i}^{{\left(\nabla_{\delta }\right)}_{+1}}\left(r,\omega \right)= & \sum_{q}C_{qi}^{{\left(\nabla_{\delta }\right)}_{+1}}\left(\omega \right)\chi_{q}\left(r\right), \end{array}$$

where $C_{qi}^{\left(0\right)}$, $C_{qi}^{{\left({\left[r\times \nabla \right]}_{\beta }\right)}_{0}}\left(\omega \right)$, $C_{qi}^{{\left(r_{\beta }\right)}_{0}}\left(\omega \right)$, $C_{qi}^{{\left({\left[r\times \nabla \right]}_{\beta }\right)}_{-1}}\left(\omega \right)$, $C_{qi}^{{\left(\nabla_{\beta }\right)}_{-1}}\left(\omega \right)$, $C_{qi}^{{\left({\left[r\times \nabla \right]}_{\beta }\right)}_{+1}}\left(\omega \right)$ and $C_{qi}^{{\left(\nabla_{\delta }\right)}_{+1}}\left(\omega \right)$ are expansion coefficients. The superscript (0) indicates canonical unperturbed coefficients, whereas

(77) $$C_{qi}^{{\left({\left[r\times \nabla \right]}_{\beta }\right)}_{0}}\left(\omega \right)=\sum_{m}^{\mathrm{vir}}\left[\sum_{j\neq a}\frac{\langle j|\sum_{i}\epsilon_{\beta \gamma \delta }r_{i\gamma }\nabla_{i\delta }|a\rangle }{\omega_{ja}^{2}-\omega^{2}}S_{im,j}\right]C_{qm}^{\left(0\right)}$$

(78) $$C_{qi}^{{\left(r_{\beta }\right)}_{0}}\left(\omega \right)=\sum_{m}^{\mathrm{vir}}\left[\sum_{j\neq a}\frac{\langle j|{\hat{r}}_{\beta }|a\rangle }{\omega_{ja}^{2}-\omega^{2}}T_{im,j}\right]C_{qm}^{\left(0\right)}$$

(79) $$C_{qi}^{{\left({\left[r\times \nabla \right]}_{\beta }\right)}_{-1}}\left(\omega \right)=\sum_{m}^{\mathrm{vir}}\left[\sum_{j\neq a}\frac{\langle j|\sum_{i}\epsilon_{\beta \gamma \delta }r_{i\gamma }\nabla_{i\delta }|a\rangle }{\omega_{ja}\left(\omega_{ja}^{2}-\omega^{2}\right)}T_{im,j}\right]C_{qm}^{\left(0\right)}$$

(80) $$C_{qi}^{{\left(\nabla_{\beta }\right)}_{-1}}\left(\omega \right)=\sum_{m}^{\mathrm{vir}}\left[\sum_{j\neq a}\frac{\langle j|\sum_{i}\nabla_{i\beta }|a\rangle }{\omega_{ja}\left(\omega_{ja}^{2}-\omega^{2}\right)}T_{im,j}\right]C_{qm}^{\left(0\right)}$$

(81) $$C_{qi}^{{\left({\left[r\times \nabla \right]}_{\beta }\right)}_{+1}}\left(\omega \right)=\sum_{m}^{\mathrm{vir}}\left[\sum_{j\neq a}\omega_{ja}\frac{\langle j|\sum_{i}\epsilon_{\beta \gamma \delta }r_{i\gamma }\nabla_{i\delta }|a\rangle }{\omega_{ja}^{2}-\omega^{2}}T_{im,j}\right]C_{qm}^{\left(0\right)}$$

(82) $$C_{qi}^{{\left(\nabla_{\delta }\right)}_{+1}}\left(\omega \right)=\sum_{m}^{\mathrm{vir}}\left[\sum_{j\neq a}\frac{\omega_{ja}\langle j|\sum_{i}\nabla_{i\delta }|a\rangle }{\omega_{ja}^{2}-\omega^{2}}T_{im,j}\right]C_{qm}^{\left(0\right)}$$

are frequency dependent perturbed coefficients. In our implementation, transition amplitudes $S_{j}$ and $T_{j}$ and corresponding transition energies $\omega_{ja}$ are obtained by means of a TD–HF≡RPA (or TD–DFT ) calculation. The full procedure for computing frequency dependent MEMDP densities has been implemented within the freely available SYSMOIC program package [59].

## 8. Results and Discussion

The large quantity of material exposed in the previous sections must be handled carefully to avoid misunderstanding, by making appropriate choices for MEMDPs to be calculated and for systems to be considered. At the current stage of the art, we have decided to restrict the compounds to only one molecule, that is, hydrogen peroxide, focusing on the *average values* of MEMDP tensors and densities, which are connected to one of the most important chiral properties, that is, the specific optical rotatory power, briefly specific rotation.

Calculations have been performed on the $R_{a}$ enantiomer of $H_{2}O_{2}$, studying MEMDPs as a function of the dihedral angle, at the time-dependent Hartree–Fock (TD–HF) level of theory, adopting a fairly large basis set consisting of the uncontracted d-aug-cc-pVQZ [60,61,62] on hydrogen atoms and d-aug-cc-pVTZ [60,61,62] on oxygen atoms. Basis sets have been downloaded from BSE [63,64]. The specific rotation for the various formalisms, as well as the related density functions, have been calculated at the wavelengths of 355 and 633 nm, which are commonly used in cavity ring-down polarimetry (CRDP) experiments [65,66], and at the sodium D-line wavelength of 589.3 nm, using the formula [15]

(83) $${\left[\alpha \right]}_{\lambda }=\frac{28800\pi^{2}N_{A}}{\lambda^{2}M}\beta ,$$

which provides the specific rotation in the usual deg [dm g/${\mathrm{cm}}^{3}]{}^{-1}$ units, when the radiation wavelength λ is in cm, the molecular mass *M* in g mol${}^{-1}$ and $\beta =-\mathrm{Tr}(\kappa^{\prime })/\left(3\omega \right)$ in ${\mathrm{cm}}^{4}$.

**Figure 1:**
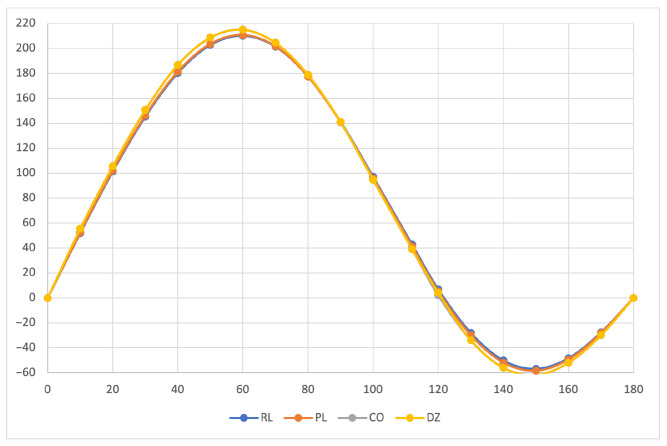
Specific rotation of the $R_{a}$ enantiomer of the hydrogen peroxide molecule, calculated at TDHF level of theory with the larger basis set, according to Equation (83) for λ=355 nm, as a function of the dihedral angle for the MEMDP defined in Equation (18) for (R,L) and (P,L) formalisms, and the MEMDP defined in Equations (37) and (50) using the common origin (CO) and CTOCD–DZ (DZ) methods to compute the current density tensor, respectively.

**Figure 2:**
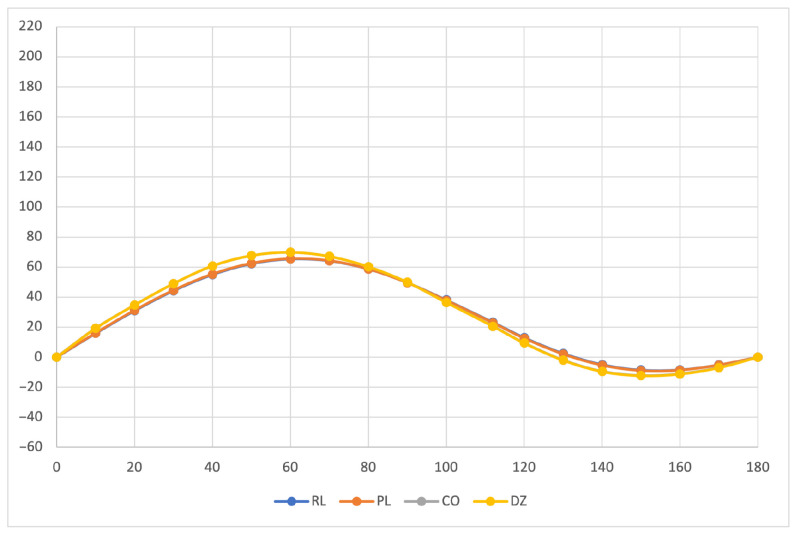
Specific rotation at λ=589.3 nm. For other details see caption to Figure 1.

**Figure 3:**
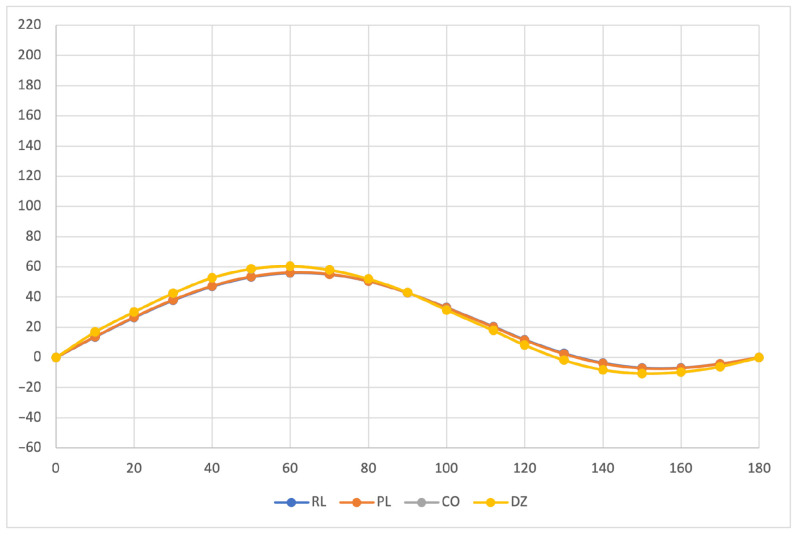
Specific rotation at λ=633 nm. For other details see caption to Figure 1.

The calculated specific rotation as a function of the dihedral angle is reported in Figure 1, Figure 2 and Figure 3 for all the formalisms here adopted for the determination of MEMDPs. Owing to the high quality of the basis set employed, only very small deviations can be observed when comparing the results arrived at via different gauges. In agreement with previous reports [67,68], the specific rotation depends strongly on the radiation frequency and it changes sign at about 120${}^{\circ }$ for λ=355 nm and at about 130${}^{\circ }$ for the other two wavelengths.

The very good agreement between length and velocity gauges, that is, (R,L) and (P,L) formalisms, is consistent with nearly origin-independent (R,L) specific rotations. The same can also be claimed for the specific rotations determined by CDTs calculated by the common origin approach, which are virtually coincident with those obtained using the origin-independent CTOCD–DZ method.

**Figure 4:**
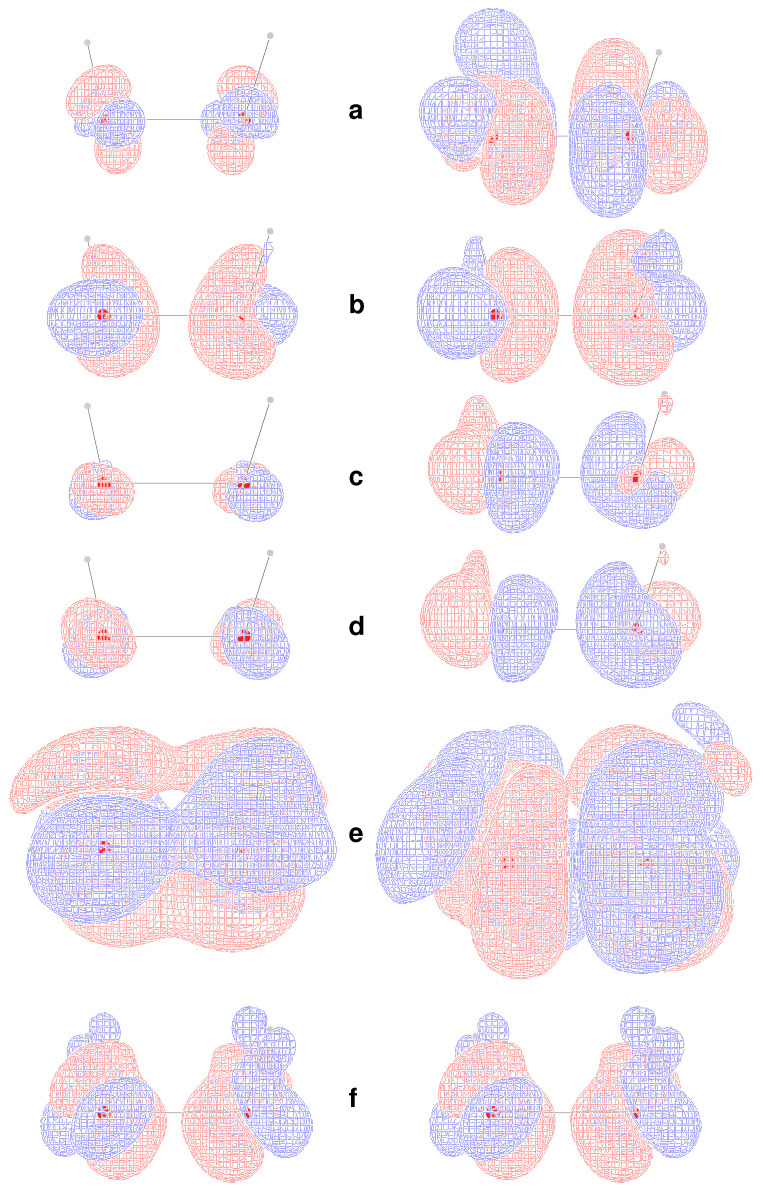
Calculated MEMDP densities at TDHF/6-31G(d,p) level of approximation, plotted as iso-value surfaces: red +200, blue −200 deg [dm g/${\mathrm{cm}}^{3}]{}^{-1}a_{0}^{-3}$. All densities on the **left**/**right** are for the ‘000’/‘123’ origin, see text. Labels (**a**–**f**) denote the six different densities as summarized in Table 1.

In principle, origin-independence cannot be easily inferred *a priori* for any of the MEMDP density functions introduced in Section 7, even if the related specific rotations turn out to be virtually invariant of the origin upon integration. This is quite an interesting point, since translational invariance is a fundamental requirement for any physically meaningful density, *irrespective of basis set choice*. To investigate this aspect, we have calculated the densities (58)–(63) for two different origins, adopting the rather small 6-31G(d,p) basis set. One origin, hereafter denoted ‘000’, has been chosen by making it coincide with the center of positive charges. The second origin, hereafter referred to as ‘123’, has been set shifting the previous one by 1, 2, and 3 bohr along *x*, *y*, and *z*, respectively. Results are shown in Figure 4, where the six densities have been plotted as signed iso-surfaces, side by side for the two origins: ‘000’ on the left; ‘123’ on the right; red/blue positive/negative.

As can be observed, the first five densities with label **a**–**e** show a marked origin-dependence. The last one, corresponding to the CTOCD–DZ CDT, is clearly origin-independent with respect to both passive and active translations, that is, translation of the origin of angular momentum and translation of the molecule as a whole. This result is very appealing, also in consideration of the fact that it is independent of basis set quality. Some more points of interest are:

1. Densities **a** and **c** are all different, but yield the same $\kappa_{\alpha \alpha }^{\prime }$ value for the same origin upon integration, irrespective of basis set, whereas computations corresponding to different origins provide the same value only in the limit of complete basis set;
2. Densities **b** and **d** integrate for both origins to the same $\kappa_{\alpha \alpha }^{\prime }$ basis set dependent value, which converges to the same optical rotation given by densities **a** and **c** in the complete basis set limit;
3. Density **e** depends on the origin—the related optical rotation does not, but improves by increasing the basis set quality toward the complete basis set result;
4. As already remarked, density **f** is origin-independent—the related optical rotation equals that obtained from density **e**.

Therefore, on the basis of the results discussed above, the MEMDP density that we recommend, as the one providing the most reliable physical interpretation, is that connected to the CTOCD–DZ current density.

Going back to the variation of optical rotation as a function of the $H_{2}O_{2}$ dihedral angle, we have found it interesting to study how MEMDP density varies with respect to internal rotation. For this purpose, we have calculated the optical rotation density in the DZ formalism for a selection of dihedral angles at a wavelength of 355 nm. Looking at Figure 1, five dihedral angles are of interest, that is, the two at which the optical rotation is at the maximum ($\delta ∼60^{\circ }$) and at the minimum ($\delta ∼150^{\circ }$) and the angle at which optical rotation changes sign ($\delta ∼120^{\circ }$), in addition to $\delta =0^{\circ }$ and $\delta =180^{\circ }$, corresponding to $C_{2v}$ and $C_{2h}$ structures, respectively.

**Figure 5:**
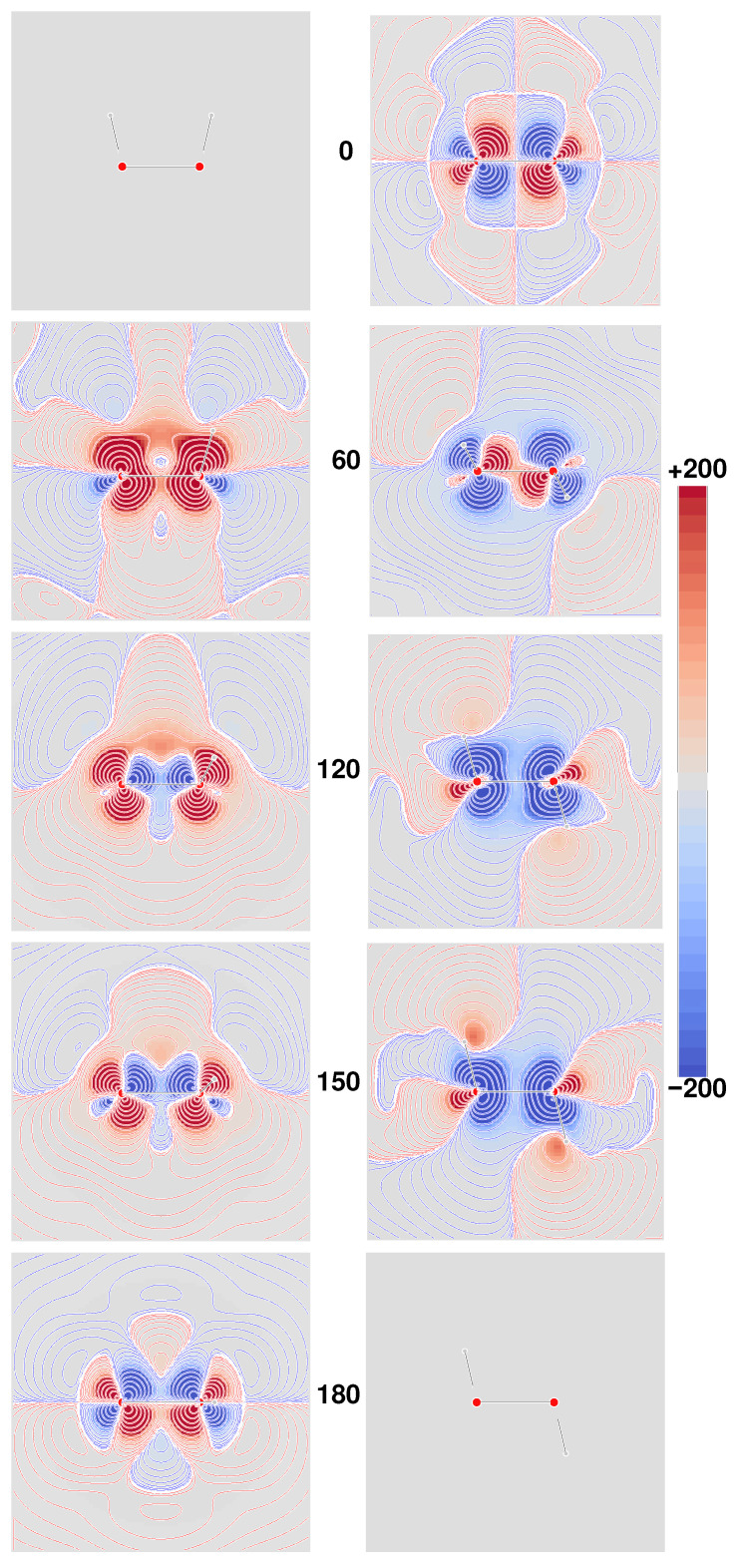
Origin-independent CTOCD–DZ MEMDP densities calculated at 355 nm using TDHF theory and the largest basis set, displayed for five different values of the $H_{2}O_{2}$ dihedral angle. On the left, the plotting plane contains the main symmetry axis; on the right, the plane is perpendicular to the $C_{2}$ symmetry axis. Each plotting area is a square centered in the O–O bond midpoint, with a side of 10 $a_{0}$. Side bar values are in deg [dm g/${\mathrm{cm}}^{3}]{}^{-1}a_{0}^{-3}$.

Computed optical rotation densities for the selected δs are shown as diverging color maps [69] in Figure 5, for planes containing the oxygen atoms, parallel (left) and perpendicular (right) to the $C_{2}$ symmetry axis. Positive/negative density values are red/blue. As can be observed, the specific rotation density is mainly located in the vicinity of oxygen atoms, with a conspicuous alternation of the sign.

**Figure 6:**
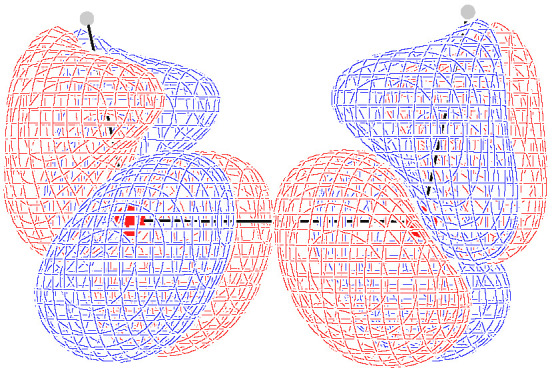
Calculated CTOCD–DZ MEMDP density at TDHF level using the largest basis set, plotted as iso-value surfaces: red +200, blue −200 deg [dm g/${\mathrm{cm}}^{3}]{}^{-1}a_{0}^{-3}$ for the hydrogen peroxide in the $C_{2v}$ point group symmetry.

**Figure 7:**
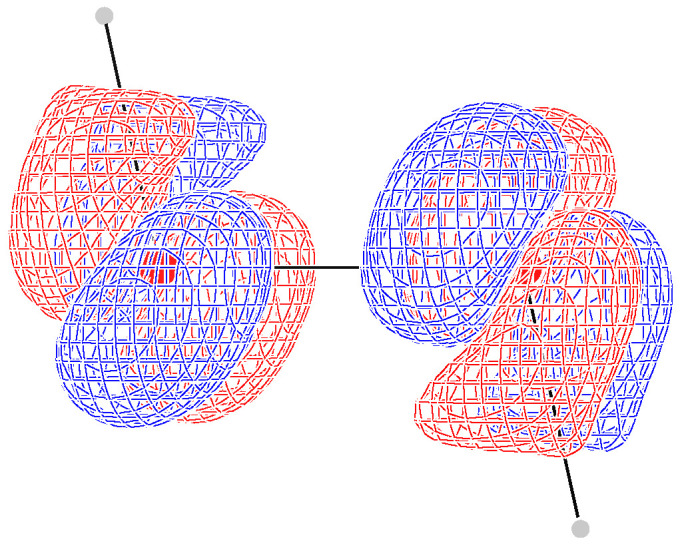
Calculated CTOCD–DZ MEMDP density for the hydrogen peroxide in the $C_{2h}$ point group symmetry. For other details see Figure 6 caption.

At $\delta =0^{\circ }$ and $\delta =180^{\circ }$, the symmetry of the density maps is consistent with the obviously vanishing optical rotation. In particular, the specific rotation density changes sign by reflection through a symmetry plane and vanishes at all its points. This is the typical feature of the scalar product between a polar vector and an axial vector, as it can be easily recognized, taking the traces of any of the $k^{\prime }$ tensors. For $\delta =0^{\circ }$, two such planes are present, that is, $\sigma_{v}$ and $\sigma_{v}^{\prime }$; for $\delta =180^{\circ }$ only $\sigma_{h}$ is present. Figure 6 and Figure 7 show this feature quite clearly for $\delta =0^{\circ }$ and $\delta =180^{\circ }$, respectively. For all the intermediate conformers, the absence of symmetry planes gives rise to positive and negative regions which do not cancel one another out. At $\delta =60^{\circ }$, a red (positive) contribution is dominant over the plane containing the $C_{2}$ axis; at $\delta =150^{\circ }$ a blue (negative) contribution over the plane perpendicular to the $C_{2}$ axis prevails; at $\delta =120^{\circ }$, the two slices in Figure 5 suggest how all contributions cancel out, passing from positive to negative optical rotation.

In summary, as far as we can see from this simple example, the specific rotation density provided by the CDT evaluated by the CTOCD–DZ approach is a function characterized by high intensity peaks of the opposite sign in the proximity of atoms, whose symmetry is clearly connected with the integrated property. It enables us to understand more precisely how the absence of symmetry planes gives rise to optical rotation.

Nonetheless, it remains to be understood how the dominant sign of optical rotation density is connected with the molecular configuration, which implies that further investigations are needed in this regard.

## 9. Concluding Remarks and Outlook

The response of a molecule to a beam of light, represented for the sake of simplicity as a monochromatic plane wave, has been formulated in terms of oscillating polarization of the electronic distribution induced by time derivative of the electric field associated to the radiation, together with the electronic current density induced by the oscillating magnetic field. It has been shown that this oscillating polarization can be related to several spatial densities of the mixed electric dipole–magnetic dipole polarizability, whose trace is connected with the specific optical rotation power of a chiral molecule.

Six different MEMDP densities have been defined and their features have been carefully investigated along with their implementation in a computer package. Origin-dependence has been checked for passive and active translations of the coordinate system and for a change of the origin of the electronic angular momentum. Only the specific rotation density, defined via the dynamic electronic current computed within the CTOCD–DZ procedure, was found to be origin-independent.

The trace of the CTOCD–DZ current density tensor has been studied as a function of the dihedral angle of a simple model system, the hydrogen peroxide molecule. Visualizations of molecular domains, which mainly determine optical rotatory power, have been reported.

Future studies are needed to connect the sign of the density function to the molecular configuration. Further, the study of the optical rotation of oriented molecules in ordered phase seems within reach via the off-diagonal components of current density tensors evaluated by the CTOCD–DZ approach to magnetic-field induced dynamic current density. The representation of streamline and the modulus of components of the dynamic current density in various spatial directions is presently being developed.

## Figures and Tables

**Table 1:** Main equations used to define and compute the MEMDP densities for the six formalisms considered here.

Figure 3	Tensor	Defining	Operative
Labels	Formalisms	Equations	Equations
**a**	$k_{\alpha \alpha }^{\prime (r,L)}\left(r,\omega \right)$	(21) and (58)	(64)
**b**	$k_{\alpha \alpha }^{\prime (p,L)}\left(r,\omega \right)$	(28) and (60)	(66)
**c**	$k_{\alpha \alpha }^{\prime (l,r)}\left(r,\omega \right)$	(59)	(65)
**d**	$k_{\alpha \alpha }^{\prime (l,P)}\left(r,\omega \right)$	(29) and (61)	(67)
**e**	$-\frac{1}{\omega }J_{\alpha }^{B_{\alpha }}\left(r,\omega \right)$	(23) and (37)	(68)
**f**	$-\frac{1}{2\omega }I_{\alpha }^{B_{\alpha }}\left(r,\omega \right)$	(50)	(69)

## Data Availability

The data that support the findings of this study are available from the corresponding author upon reasonable request.

## Sample Availability

Samples of the compounds are not available from the authors.
